# Effects of Eggshell Calcium- and Vitamin D-Fortified HMR Combined with Aerobic Exercise on Bone Mineral Density in Postmenopausal Women: A Pilot Randomized Controlled Trial

**DOI:** 10.3390/nu18040605

**Published:** 2026-02-12

**Authors:** Susie Jung, So-Hui Shin, Yoon-Suk Kim, Nam-Seok Joo, Kyung-Jin Yeum

**Affiliations:** 1 Department of Family Practice and Community Health, Ajou University School of Medicine, Suwon 16499, Republic of Korea; lovehrh@naver.com; 2 Department of Food and Nutrition, College of Biomedical and Health Science, Konkuk University, Chungju 27478, Republic of Korea

**Keywords:** postmenopause, bone density, calcium, vitamin D, dietary supplements, exercise

## Abstract

Background/Objectives: Adequate calcium and vitamin D intake, along with regular physical activity, is essential for maintaining skeletal health in postmenopausal women. In populations with low dairy consumption, sustainable and bioavailable calcium sources are required to support bone integrity. Eggshell powder offers a highly bioavailable, eco-friendly, and cost-effective calcium source consistent with environmental sustainability goals. This pilot randomized, double-blind, controlled trial investigated the effects of calcium- and vitamin D-fortified home meal replacements (HMRs) made with eggshell powder, combined with weight-bearing aerobic exercise, on bone mineral density (BMD) and bone turnover markers in postmenopausal women. Methods: Thirty-six women aged 50–59 years were randomly assigned (1:2 ratio) to a control group (regular HMR) or an intervention group (HMR fortified with 418 mg eggshell-derived calcium and 837 IU vitamin D). Participants consumed one HMR daily, five days per week, and were encouraged to engage in weight-bearing aerobic exercise for 30–60 min, five times weekly, over six months. Results: High adherence was observed for both dietary (94%) and exercise regimens. No significant differences were found between groups in overall BMD or bone metabolic markers after six months. Exploratory post hoc subgroup analysis suggested possible exercise–nutrition interactions in lumbar spine BMD. Conclusions: No significant overall BMD effect was found. Fortified HMRs achieved excellent adherence and corrected nutrient deficiencies, demonstrating feasibility. Future large-scale trials with adequate power based on realistic effect sizes are warranted to evaluate reproducibility of these exploratory findings.

## 1. Introduction

Bone health is a major concern for postmenopausal women, as estrogen deficiency after menopause accelerates bone resorption and suppresses bone formation, leading to reduced bone mineral density (BMD) and an increased risk of fractures [1]. Given these physiological vulnerabilities, osteoporosis has emerged as a significant global public health issue, particularly among postmenopausal women, due to its strong association with fractures, morbidity, mortality, and substantial socioeconomic burden [2,3]. In Asian populations, including Korea, the prevalence of osteoporosis is notably high; approximately 38% of postmenopausal Korean women are affected [4]. Among the various risk factors for osteoporosis, modifiable lifestyle factors—particularly insufficient calcium and vitamin D intake, as well as inadequate physical activity—are of special importance, alongside other established factors such as advanced age and low body mass index (BMI) [5]. In Korea, calcium and vitamin D intakes remain persistently inadequate, largely due to low dairy consumption associated with traditional dietary patterns and widespread lactose intolerance, and this insufficiency becomes increasingly pronounced with advancing age [6,7]. Furthermore, physical inactivity exacerbates bone loss, as the absence of regular weight-bearing or resistance exercise contributes to decreased BMD and an elevated risk of osteoporosis [8].

Adequate intake of calcium and vitamin D is critical for maintaining BMD and reducing fracture risk. Numerous randomized controlled trials and meta-analyses have demonstrated that combined calcium and vitamin D supplementation improves BMD at key skeletal sites and lowers the incidence of hip fractures in postmenopausal women [9]. Nevertheless, adherence to supplementation or fortified food intake remains suboptimal. To enhance compliance and effectiveness, various food-based delivery systems—such as dairy products and plant-based alternatives like fortified soymilk—have been developed [10,11]. Expanding the availability of calcium-fortified foods may be an effective approach to improving BMD and achieving optimal peak bone mass, especially in individuals with limited dairy consumption due to lactose intolerance or personal preference. Food fortification, particularly of widely consumed products, is increasingly recognized as a practical and sustainable strategy to promote bone health across populations [12,13].

In addition to nutritional interventions, physical activity—particularly weight-bearing aerobic exercise and resistance training—plays a vital role in maintaining and enhancing bone health. These exercises improve BMD, modulate bone turnover markers, and positively influence hormonal balance, thereby reducing bone loss in postmenopausal women [14,15]. The World Health Organization (WHO) recommends at least 150 min of moderate-intensity or 75 min of vigorous-intensity aerobic activity weekly, along with muscle-strengthening exercises on two or more days [16]. However, physical inactivity remains highly prevalent, affecting nearly one in four adults globally [17], and sedentary behavior continues to pose a major public health challenge globally, including in Korea [18]. A combination of aerobic and resistance exercise is regarded as the most effective approach for improving overall BMD, and aerobic exercise alone has been shown to be a particularly potent stimulus for improving lumbar spine BMD [19]. Mechanical loading directly stimulates the bone matrix by activating mechanotransduction pathways in osteocytes, which promotes osteoblast activity and reduces sclerostin expression. It also indirectly modulates bone remodeling through anti-inflammatory mechanisms and myokine-mediated signaling [20]. On a microscopic level, aerobic exercise can mitigate the physiological consequences of estrogen deficiency, thereby supporting osteoblast activity while suppressing osteoclast activity [21]. In addition, it contributes to protecting against bone loss by regulating trace element homeostasis and enhancing antioxidant defense system [22]. Therefore, an integrated approach combining adequate calcium and vitamin D intake with regular aerobic and resistance exercise is essential to preserve bone health [8,23].

As Korea transitions into a super-aged society, home meal replacements (HMRs) have emerged as a practical vehicle for nutrient delivery [24]. Fortifying HMRs with eggshell calcium—a highly bioavailable source compared to calcium carbonate—and vitamin D helps support consistent daily intake, which is important for maintaining the calcium-phosphate balance and suppressing parathyroid hormone (PTH) and bone turnover markers [25]. Clinical evidence suggests that such fortified food vehicles can be comparable to conventional supplements in improving vitamin D status and modulating bone turnover markers in postmenopausal women [26].

Given these challenges, culturally appropriate, effective, and sustainable strategies are needed to prevent bone loss in postmenopausal women, particularly among those with low dairy intake and insufficient physical activity. Fortifying commonly consumed foods, such as HMRs, with natural calcium sources (e.g., eggshell powder) and vitamin D—alongside simple, daily exercise recommendations—may represent a feasible approach to improving bone health in this vulnerable population. In a previous study, we developed an HMR fortified with eggshell powder and vitamin D that reflected the dietary preferences of Korean adults and demonstrated beneficial effects on bone health in postmenopausal women [27].

Therefore, we hypothesized that a 6-month intervention with eggshell calcium- and vitamin D-fortified HMR, combined with regular aerobic exercise, would more effectively attenuate bone loss and improve bone metabolism markers compared to standard HMR. To test this hypothesis, we conducted a pilot randomized controlled trial in postmenopausal women, who were encouraged to maintain a consistent aerobic exercise, comparing the effects of fortified HMR versus standard HMR on bone mineral density (BMD) and bone-related markers. In addition, we pre-planned an exploratory subgroup analysis to examine whether the bone-related responses to the fortified HMR differed according to participants’ actual exercise volume and adherence levels. This pilot efficacy trial had dual objectives: (1) feasibility assessment of fortified HMR adherence/safety, and (2) preliminary testing of large-effect reproducibility from prior nutrition trials in a combined nutrition–exercise context.

## 2. Materials and Methods

### 2.1. Study Design

This randomized, double-blind, controlled, parallel-group trial was conducted in postmenopausal women. Participants were stratified by age (50–55 and 56–60 years) and randomly assigned in a 1:2 ratio to either (1) a control group consuming a regular Home Meal Replacement (HMR) containing sweet pumpkin powder (600 mg/serving) or (2) an intervention group consuming an HMR fortified with eggshell powder (418 mg calcium/serving) and vitamin D (21 μg or 837 IU/serving) and containing tomato powder (600 mg/serving). The 1:2 allocation ratio was employed to maximize the amount of efficacy and safety data collected for the fortified HMR intervention and to allow for exploratory subgroup analyses based on exercise status, while still maintaining adequate statistical power within the constraints of a pilot study. Sweet pumpkin and tomato powders were added exclusively as marker ingredients to verify compliance and confirm group assignment; aside from these marker components, the HMR formulations were identical between groups. Although the inclusion of these powders could result in slight differences in color or taste, the identity of which powder corresponded to which group was concealed from both participants and investigators, thereby maintaining blinding throughout the study. Randomization codes were generated and maintained by an independent clinical trial center staff member.

**Figure 1:**
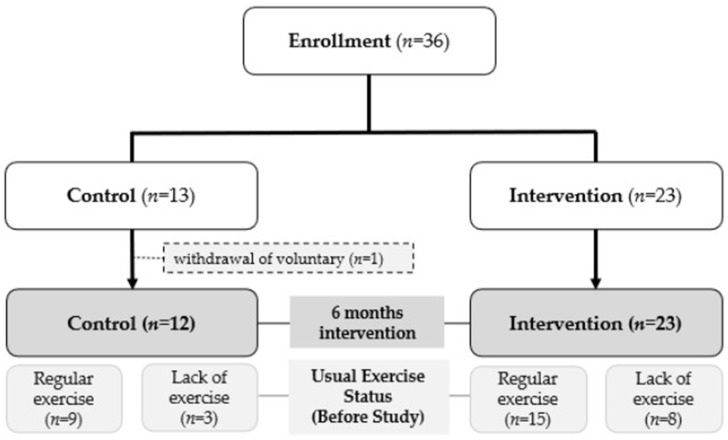
Study Participant Enrollment and Study Flow. Flow diagram illustrating participant recruitment, screening, eligibility assessment, allocation to control and intervention groups, follow-up, and inclusion in the final analysis.

In this trial, the HMR product was based on a commercially available Yulmu tea powder. For the control group, sweet pumpkin powder was incorporated into the Yulmu tea, whereas the treatment group received Yulmu tea fortified with eggshell powder, vitamin D, and tomato powder as marker ingredients. All participants consumed one serving of their assigned HMR per day, 5 days per week, prepared by mixing the powder with water and ingesting it in the form of a tea-like snack beverage, while maintaining their usual dietary habits for all other meals. They were also encouraged to perform weight-bearing aerobic exercise for 30–60 min/session at least 5 times per week. Exercise type was not restricted; recommended activities included walking, light jogging, hiking on maintained trails, aerobics, and dance. Dietary and exercise adherence were monitored using daily food diaries and exercise logs. The study flow diagram is shown in Figure 1.

The study was approved by the Institutional Review Board of Ajou University Hospital (AJOUIRB-IV-2023-292). Written informed consent was obtained from all participants. No important changes to the study methods or eligibility criteria occurred after trial commencement.

### 2.2. Sample Size

Sample size was estimated using our previous clinical trial [27] and a network meta-analysis of exercise interventions in postmenopausal women [28]. Although previous trial employed a different type of HMR and intake format, it provided an appropriate reference because the HMR was similarly fortified with eggshell-derived calcium and vitamin D and the participants were of a comparable age range. An a priori power analysis (α = 0.05, 80% power, two-tailed *t*-test) using Cohen’s d = 1.10—derived from the prior trial (d = 0.97) and supported by network meta-analysis SMDs of 0.17–1.40—with 2:1 allocation and 10% dropout yielded enrollment of 23 treatment and 13 control participants (total 36). This optimistic estimation was used to test reproducibility of the large effects observed in earlier nutritional pilot trials within the context of the current combined nutrition and exercise intervention, particularly under fixed budgetary constraints. We acknowledge that this approach renders the study likely underpowered for detecting the moderate effects (d = 0.2–0.5) typically observed in nutritional interventions. Completers: 23/12 (actual dropout: 2.8%). This sample size was adequate for this pilot efficacy trial’s dual objectives: establishing feasibility and testing reproducibility of previously observed effects. Future confirmatory trials should be powered for moderate effects (d = 0.5) with balanced allocation.

### 2.3. Interventions

Participants consumed one serving (approximately 20 g powder) of their assigned HMR daily, 5 days per week, for 6 months. The HMR was prepared by dissolving the powder in 200–250 mL hot water (80–90 °C) to form a tea-like beverage, consumed as a mid-morning or afternoon snack while maintaining usual diets for other meals. Control group HMR contained Yulmu tea base with sweet pumpkin powder (600 mg/serving) as compliance marker; intervention HMR contained identical Yulmu tea base fortified with eggshell powder (1.0–1.7% *w*/*w*, providing 418 mg elemental calcium/serving), vitamin D (21 μg/serving), and tomato powder (600 mg/serving) as compliance marker. Products were manufactured under Good Manufacturing Practice standards, packaged in identical blinded sachets (labeled only with participant ID and date), stored at room temperature, and dispensed monthly by study coordinators with adherence reinforced via weekly phone check-ins and returned sachet counts. Concurrently, all participants maintained weight-bearing aerobic exercise (30–60 min/session, ≥5 sessions/week; e.g., walking, jogging, hiking, aerobics) logged daily.

### 2.4. Exercise Intervention and Monitoring

Participants were encouraged to perform weight-bearing aerobic exercises, such as brisk walking, light jogging, hiking on paved trails, or aerobics, for 30–60 min per session at least five times per week. Although participants selected their preferred type of exercise to support long-term adherence, the recommended frequency and duration were standardized. To monitor exercise dose, participants received an exercise log at each visit, in which they recorded the type and duration (in minutes) of every session. These logs were collected and reviewed by the investigators at each follow-up visit to verify adherence. Based on the completed logs, all participants maintained at least five sessions per week, with each session lasting more than 30 min, throughout the study period.

### 2.5. Outcomes

Primary outcome was change in BMD from baseline to 6 months, measured by dual-energy X-ray absorptiometry (DXA). Secondary outcomes included changes in urine *N*-terminal propeptide of type I collagen (NTx), serum osteocalcin, 25-hydroxyvitamin D (25(OH)D), serum calcium, and urine calcium/creatinine (Ca/Cr) ratio at 6 months. These were prespecified in the trial protocol with no post-commencement modifications.

### 2.6. Study Population

Participant recruitment began on 20 October 2023, and follow-up for the last enrolled participant was completed on 20 October 2025. Each participant underwent registration, randomization, baseline assessment, and final follow-up evaluation at 6 months from baseline assessment. The trial proceeded to planned completion without early termination. A total of 36 postmenopausal women were enrolled; 1 participant withdrew consent after randomization. Thus, 35 participants completed the 6-month intervention (12 control; 23 intervention). All statistical analyses were conducted using per-protocol (PP) analysis, excluding the one participant who withdrew consent.

Women who visited the Department of Family Medicine outpatient clinic or the Health Promotion Center at Ajou University Hospital were enrolled after receiving a sufficient explanation of the study and providing voluntary written consent. Women aged 50–59 years with natural menopause (≥12 months of amenorrhea or final menstrual period within the prior 6–12 months) and serum follicle-stimulating hormone (FSH) > 40 mIU/mL. BMI eligibility ranged from 18.5 to 30.0 kg/m^2^. Exclusion criteria included: T-score < −2.5 at the lumbar spine, femoral neck, or total hip; use of calcium, vitamin D, or hormone therapy within 3 months; AST or ALT > 120 U/L; serum creatinine > 2.0 mg/dL; thyroid dysfunction (hypo-/hyperthyroidism, use of thyroid-regulating medication after thyroid cancer, or TSH > 10 uIU/mL or < 0.15 uIU/mL); uncontrolled hypertension (>160/100 mmHg); uncontrolled diabetes (fasting glucose > 180 mg/dL or medication changes within 3 months); malignancy within the past 5 years; esophageal motility disorders; untreated hypercalcemia or hypocalcemia; chronic diseases affecting bone metabolism (e.g., chronic liver disease, alcoholism, primary hyperparathyroidism); use of medications affecting bone metabolism (e.g., systemic steroids, diuretics); and allergy to eggshell or egg products.

### 2.7. Assessment

Baseline lifestyle habits—including smoking status, alcohol intake, and habitual physical activity—were assessed through face-to-face interviews conducted by trained study nurses. Current smokers were defined as those actively smoking or with >5 pack-years of cumulative exposure. Regular alcohol use was defined as drinking ≥ 1 time per month. Weekly alcohol intake and exercise patterns over the previous 3 months were recorded.

Anthropometric measurements, including height, weight, BMI, waist circumference, blood pressure, and pulse, were collected at baseline, 3 months, and 6 months.

After an overnight fast (≥8 h), blood samples were collected at baseline and 6 months to measure lipid profile, glucose, HbA1c, insulin, calcium, osteocalcin, PTH, and 25-hydroxyvitamin D (25(OH)D). Serum 25(OH)D was determined by radioimmunoassay (DiaSorin Inc. Stillwater, MN, USA) using a γ-counter (1470 Wizard; PerkinElmer, Waltham, MA, USA). Serum PTH was measured by chemiluminescence (DiaSorin Inc. Stillwater, MN, USA). Random urine samples were analyzed for NTx, calcium, and creatinine.

Bone mineral density (primary outcome) at the lumbar spine (L1–L4), femoral neck, and total hip was measured at baseline and 6 months using DXA (DISCOVERY-W, Hologic Inc. Marlborough, MA, USA), with coefficients of variation of 1.9% for the lumbar spine and 2.5% for the femoral neck and total hip.

Dietary intake was evaluated using 3-day food records (2 weekdays and 1 weekend day) and a validated food frequency questionnaire, with nutrient analysis performed using Can-Pro software (web ver. 5.0; The Korean Nutrition Society). Exercise adherence was assessed through weekly logs documenting the frequency and duration of weight-bearing aerobic activities.

### 2.8. Statistical Analysis

Normality of continuous variables was assessed using the Shapiro–Wilk test. Variables with normal distributions (*p* > 0.05) were compared using independent *t*-tests; non-normally distributed variables (*p* < 0.05) were compared using the Wilcoxon rank-sum test. Categorical variables were compared using Fisher’s exact test. All analyses were two-sided with statistical significance set at *p* < 0.05.

Continuous variables are reported as mean ± standard deviation (SD) in tables and as median (interquartile range) in figures. BMD values and associated *p*-values were rounded to three decimals; all other values were rounded to one decimal. Analyses and visualizations were performed using R (version 4.5.0) with RStudio.

Subgroup analyses based on physical activity were pre-planned to explore the potential synergistic effects of exercise and nutritional supplementation. Although we initially intended to stratify participants by exercise adherence during the trial, the universal compliance (100%) made this approach uninformative. Moreover, the average exercise duration during the study was 253.63 ± 95.48 min/week for the entire participants, with no significant difference between the control (244.60 ± 75.18 min/week) and intervention (267.31 ± 87.92 min/week) groups (*p* > 0.05).

Given the lack of variability during the study period, we focused our analyses on baseline habitual exercise levels, which showed a clear dichotomy. Participants were therefore categorized into a ‘Regular Exercise’ (minimum 90 min/week and average 230 min/week) and a ‘Lack of exercise’ (0 min/week) groups based on their pre-study activity patterns. Subgroup analyses were pre-specified as exploratory objectives, but final categorization was redefined post hoc based on the observed bimodal data distribution of baseline exercise. No formal adjustment for multiple testing was applied; increasing Type I error risk. Thus, these analyses were conducted to generate hypotheses for future confirmatory trials rather than to provide definitive evidence of treatment–exercise interactions.

## 3. Results

### 3.1. Baseline Characteristics

At baseline, the control and intervention groups did not differ significantly in general characteristics, including age, body weight, BMI, waist circumference, and serum lipid profiles as shown in Table 1. Bone-related biochemical indices—serum osteocalcin, urinary NTx, serum 25(OH)D, calcium, and PTH—were comparable, and BMD at the lumbar spine, femoral neck, and total hip showed no between-group differences (Table 1). All participants were non-smokers, and alcohol intake patterns were similar between groups. The participants had an average age of 55 years and were approximately 5 years post-menopause. Medical history showed that 66.7% (8/12) of the control group and 52.2% (12/23) of the intervention group were taking medications for hypertension or dyslipidemia, with no significant differences between the two groups.

Regarding habitual physical activity, participants were categorized according to their pre-enrollment exercise patterns: the “Regular Exercise” group (engaging in weight-bearing aerobic exercise for minimum 90 min/week; *n* = 24) and the “Lack of exercise” group (0 min/week; *n* = 11). At baseline, 9 of 12 participants in the control group and 15 of 23 participants in the intervention group were classified into the regular exercise group.

### 3.2. Clinical and Behavioral Outcomes After the 6-Month Intervention

After 6 months of consuming the calcium–vitamin D–fortified HMR, no significant between-group differences were observed in changes in BMD or bone metabolic markers (Table S1). Dietary adherence was excellent, with participants consuming 110.3 ± 7.8 meals on average (94.0 ± 6.6% adherence), and adherence did not differ between groups (*p* = 0.236). All participants complied with the exercise recommendations. Weekly exercise time was comparable between groups (Control: 225.3 ± 86.6 min; Intervention: 268.4 ± 98.4 min; *p* = 0.149). No adverse events related to HMR consumption were reported during the study period.

### 3.3. Nutrient Intake, Stability and Biochemical Responses to the Fortified Product

**Figure 2:**
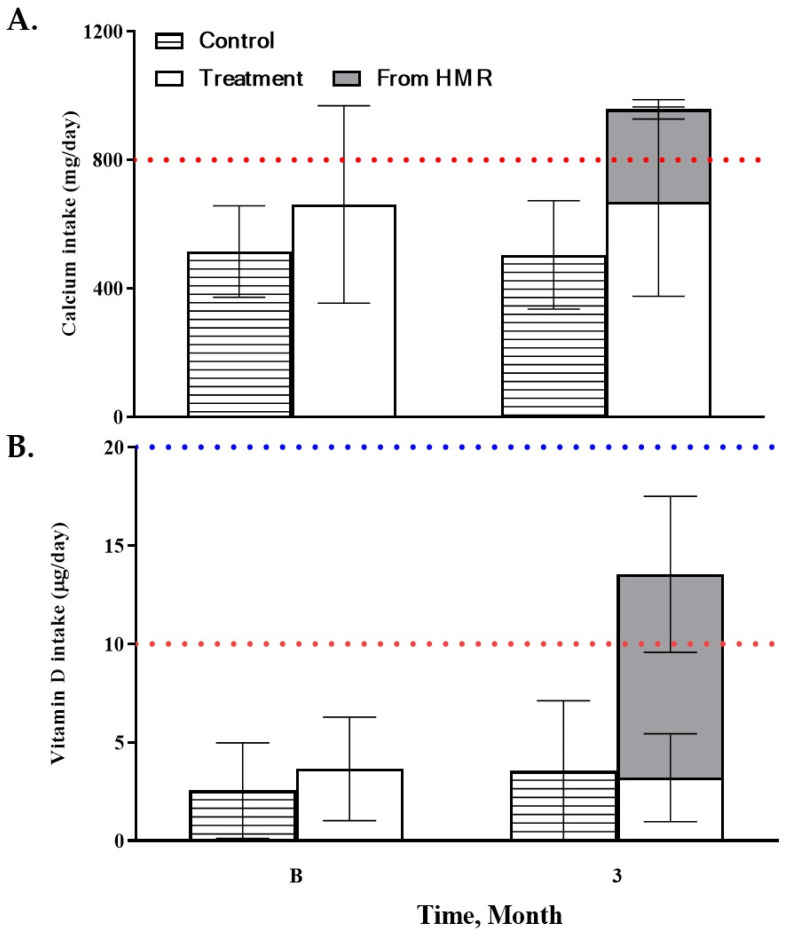
Changes in Calcium and Vitamin D Intake After the Dietary Intervention. (**A**) Daily calcium intake and (**B**) daily vitamin D intake in control (*n* = 12) and intervention groups (*n* = 23) at baseline and after the 6-month intervention. Values are presented as mean ± SD. Dietary intake was assessed using 3-day food records (2 weekdays and 1 weekend day) and analyzed with CAN-Pro software (web ver. 5.0; The Korean Nutrition Society).

At baseline, both groups exhibited insufficient calcium and vitamin D intake relative to recommended levels. Following the intervention, nutrient intake remained unchanged in the control group, whereas the intervention group showed marked increases attributable to the fortified product. Total calcium intake reached approximately 938 mg/day (Figure 2A) and vitamin D intake reached 22.7 µg/day (Figure 2B), exceeding recommended intake levels.

The stability analysis showed that calcium content in the fortified study diet remained unchanged over 18 months of storage (*p* > 0.05), while vitamin D content decreased moderately, showing an 86.7% retention rate at 18 months (Table 2).

**Figure 3:**
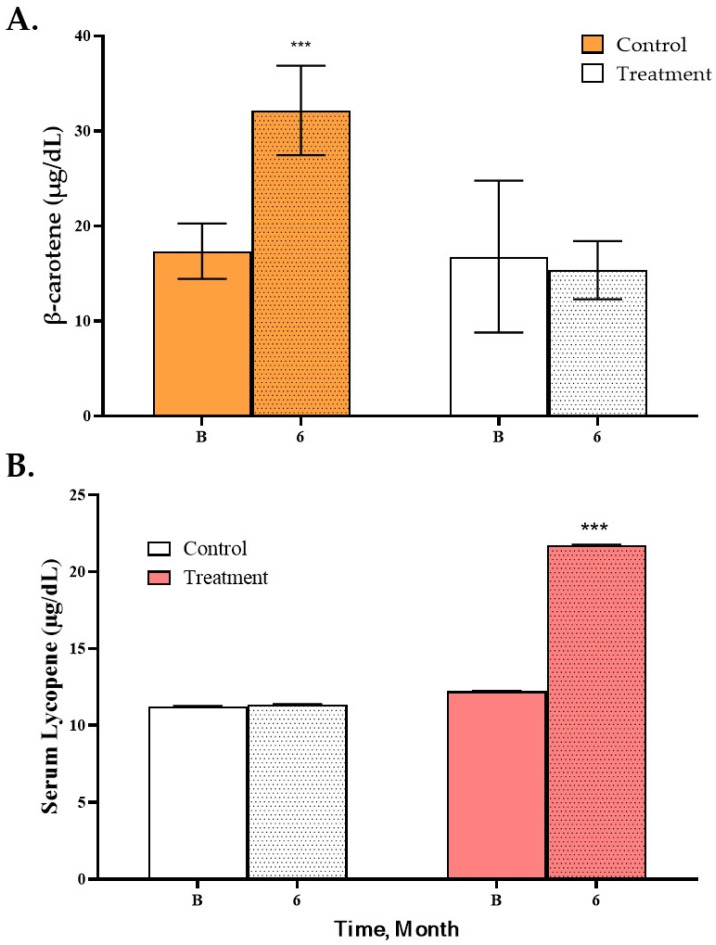
Changes in Serum β-Carotene (**A**) and Lycopene (**B**) Concentrations After the 6-month study diet intervention. Serum β-carotene (**A**) and lycopene (**B**) concentrations at baseline and after the 6-month intervention in control (*n* = 12) and intervention (*n* = 23) groups. Values are expressed as mean ± SD. Carotenoid concentrations were quantified using ultra-performance liquid chromatography (UPLC) after plasma extraction. Statistical significance (***) versus baseline is indicated as *p* < 0.001.

Serum carotenoid concentrations showed distinct product-dependent patterns. The control group—consuming pumpkin-containing products—exhibited significant increases in serum zeaxanthin, lutein, and β-carotene (Figure 3A). In contrast, only the intervention group demonstrated a significant rise in serum lycopene (12.2 → 21.8 µg/dL; *p* < 0.001), consistent with the tomato fortification of the HMR (Figure 3B).

### 3.4. Subgroup Analyses According to Baseline Exercise and Bone Status

As pre-specified in our exploratory research objective, subgroup analyses were conducted to evaluate whether baseline exercise habits or baseline bone status modified the effects of the fortified HMR intervention. In the overall study population, baseline exercise status was not associated with differences in BMD changes or most bone metabolic markers within the control group (all *p* > 0.1) as presented in Table 3. A greater reduction in urinary NTx was observed among those without prior exercise (−21.3 vs. −0.4), showing a trend toward significance (*p* = 0.063), but no other meaningful differences were identified (Table S2).

**Figure 4:**
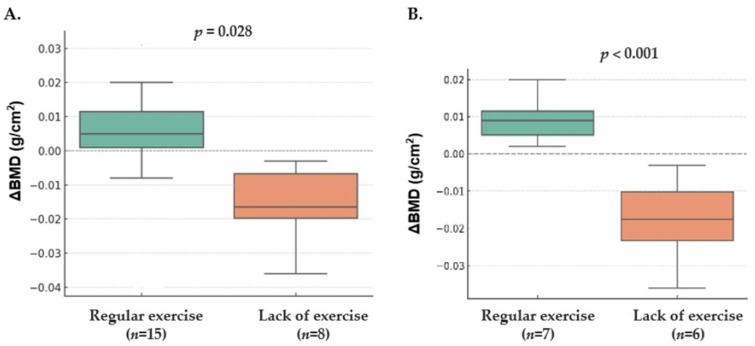
Change in Lumbar Spine BMD According to Usual Exercise Status in the entire treatment Group (**A**) and Osteopenic Participants of the treatment Group (**B**).

In contrast, among participants in the intervention group, baseline exercise habits were associated with differential changes in lumbar spine BMD. Participants who had engaged in regular weight-bearing aerobic exercise prior to the study exhibited a significant increase in lumbar spine BMD over 6 months compared with those who did not exercise (0.004 ± 0.017 vs. −0.013 ± 0.015; *p* = 0.028) as presented in Figure 4A. No group differences were observed in changes in bone metabolic markers.

Analyses restricted to participants with osteopenia showed a consistent pattern as presented in Table 4. In the control group, baseline exercise status did not influence BMD or biochemical outcomes. However, in the intervention group with osteopenia, lumbar spine BMD increased significantly among those with prior regular exercise, whereas participants without exercise showed a decline (0.009 ± 0.006 vs. −0.018 ± 0.012; *p* = 0.001) as presented in Figure 4B. Other biochemical markers, including PTH, 25(OH)D, osteocalcin, urinary NTx, and urinary calcium/creatinine ratio, did not differ between exercise categories.

Among participants with normal baseline BMD, comparisons in the control group were limited due to the small number of individuals lacking prior exercise (*n* = 1). In the intervention group with normal BMD, changes in BMD and bone metabolic markers did not differ significantly according to baseline exercise status (all *p* > 0.5).

A. Change in lumbar spine bone mineral density (BMD) over 6 months in intervention participants stratified by usual regular exercise before the study. In exploratory post hoc subgroup analysis, participants with habitual regular exercise showed a greater increase in lumbar spine BMD compared with those without regular exercise (*p* = 0.028).
B. Change in lumbar spine bone mineral density (BMD) over 6 months in osteopenic intervention participants stratified by usual regular exercise before the study. In exploratory post hoc subgroup analysis, individuals with habitual regular exercise showed a greater increase in lumbar spine BMD than those without regular exercise (*p* = 0.001; no multiplicity adjustment applied).

Regular exercise was defined as performing weight-bearing aerobic exercise for at least 90 min per week prior to the study, while lack of exercise was defined as 0 min per week.

## 4. Discussion

This pilot study evaluated the effects of a home meal replacement (HMR) fortified with eggshell-derived calcium and vitamin D on bone health in postmenopausal women. In the primary analysis, the 6-month intervention did not result in significant between-group differences in BMD or bone turnover markers. Exploratory subgroup analyses, which must be interpreted with caution due to their post hoc nature and risk of Type I error, revealed patterns indicating a possible interaction between nutritional fortification and pre-existing habitual exercise. If confirmed in larger trials, such patterns would be consistent with prior evidence demonstrating synergistic effects of combined nutritional and physical activity interventions on skeletal outcomes [9,29].

Adherence to the fortified HMR was excellent, supporting the reliability of the intervention outcomes and demonstrating the feasibility of culturally adapted fortified foods in populations with low dairy consumption. The fortified HMR substantially improved calcium and vitamin D intake, elevating both nutrients above recommended levels and effectively correcting the marked baseline insufficiency characteristic of postmenopausal Korean women. Because inadequate calcium and vitamin D intake is a modifiable determinant of age-related bone loss, this improvement represents an important achievement in nutrient adequacy, independent of short-term BMD changes. Stability assessments further confirmed that the fortified nutrients—particularly calcium—were well maintained during storage, ensuring consistent nutrient exposure over time. Biochemical markers provided supportive data regarding physiological response and confirmed product adherence. The significant increase in serum lycopene observed exclusively in the intervention group reflects effective absorption of tomato-derived carotenoids and suggests an enhancement of systemic antioxidant capacity. Given the emerging role of oxidative stress in bone remodeling [30,31], such biochemical improvements may support long-term skeletal health and confer additional cardiometabolic benefits beyond bone outcomes. Approximately two-thirds of participants reported regular exercise at baseline, which may have reduced the magnitude of between-group differences. This contrasts with studies conducted in sedentary populations, where exercise interventions or fortified foods yielded clearer improvements in BMD [15,28].

Exploratory subgroup analyses examined potential effect modification by baseline exercise habits. Participants in the intervention group who engaged in regular weight-bearing aerobic exercise prior to the study showed a slight increase in lumbar spine BMD, whereas those without exercise habits showed a decline. If replicated, this observed pattern could be biologically plausible based on prior research on exercise-nutrition interactions [32] and would align with reports of potential synergistic effects of combined nutritional and exercise interventions on trabecular-rich skeletal sites [33]. The lumbar spine, characterized by metabolically active trabecular bone, may respond more rapidly to the combined effects of nutrient fortification and habitual mechanical loading than cortical-dominant regions such as the femoral neck [34,35].

The potential association between habitual physical activity and the observed bone response, if confirmed, would align with the multifactorial nature of musculoskeletal health in postmenopausal women. Previous research has shown that regular engagement in physical activities, such as Zumba or Pilates, can improve functional performance, including lower body muscle strength and dynamic balance, which are key contributors to mechanical loading on the skeletal system [36]. Additionally, higher physical activity levels in older women are associated with more favorable body composition, reduced disability, and better management of age-related physical declines [37]. Our exploratory findings, though requiring confirmation, raise the speculative hypothesis that pre-existing exercise habits may be associated with musculoskeletal characteristics that could modulate the skeletal response to nutritional fortification with eggshell calcium and vitamin D. This potential interaction, if validated in prospective studies specifically designed to test this hypothesis, could contribute to explaining the observed preservation of lumbar spine BMD; however, given the post hoc and exploratory nature of these findings, any mechanistic interpretation remains premature.

Although weight-bearing exercise was recommended for all participants during the trial, the absence of structured or supervised protocols likely contributed to the limited intervention effect among those without established exercise habits. In contrast to supervised or structured exercise protocols that consistently improve bone metabolic markers [38,39], self-reported exercise measures in our study may also have attenuated the precision of estimates. The pattern of lumbar spine BMD preservation among individuals with habitual exercise raises the hypothesis that pre-existing physical activity may influence the skeletal response to fortified foods [40], though this requires confirmation in adequately powered trials. However, self-reported exercise logs represent a limitation, as objective monitoring (e.g., accelerometers) would provide greater precision.

The feasibility demonstrated in this study supports WHO recommendations emphasizing combined dietary fortification and physical activity for osteoporosis prevention [41]. Fortified HMRs may offer a practical, sustainable, and culturally acceptable means of improving calcium and vitamin D intake, particularly among individuals who avoid dairy products. For sedentary individuals, however, structured exercise programs that incorporate progressive resistance and moderate-to-vigorous aerobic activity may be necessary to achieve measurable skeletal benefits [42]. Longer intervention periods—typically ≥12–18 months—may also be required to detect meaningful changes in BMD [43].

This study has several limitations. First, the small sample size (*n* = 36) and the power calculation based on a large effect size (Cohen’s d = 1.10)—derived from our previous work to test reproducibility under budgetary constraints— rendered the trial underpowered to detect moderate effects typically seen in BMD interventions; thus, the lack of significance in the primary outcome must be interpreted acknowledging this limitation. Second, the subgroup analyses were refined post hoc based on the observed data distribution, which substantially increases the risk of Type I error and may reflect chance findings; accordingly, these findings must be viewed as hypothesis-generating rather than confirmatory. Third, physical activity was assessed via self-reported logs rather than objective measures like accelerometers, potentially introducing ambiguity regarding exercise intensity. Despite these constraints, high adherence and nutritional improvements provide feasibility data for larger trials with objective exercise monitoring.

## 5. Conclusions

This pilot trial found no significant BMD differences between the fortified HMR and control groups. However, it achieved excellent adherence and corrected key nutrient deficiencies, establishing feasibility for culturally appropriate fortification strategies in postmenopausal women. Future adequately powered trials with prospective exercise stratification are needed to explore the potential synergistic effects suggested by these exploratory, hypothesis-generating findings.

## Figures and Tables

**Table 1:** Baseline characteristics of the study participants.

Variables	Total (*n* = 35)	Control (*n* = 12)	Intervention (*n* = 23)	*p*-Value
**General characteristics**				
Age (years)	54.6 ± 2.6	55.0 ± 2.6	54.4 ± 2.6	0.545
Body Weight (kg)	55.2 ± 6.6	54.3 ± 6.5	55.6 ± 6.7	0.434
Height (cm)	157.0 ± 4.2	156.6 ± 3.1	157.2 ± 4.7	0.671
BMI (kg/m^2^)	22.4 ± 2.4	22.1 ± 2.3	22.5 ± 2.5	0.664
Waist circumference (cm)	80.2 ± 6.2	78.5 ± 5.2	81.0 ± 6.6	0.222
Systolic BP (mmHg)	118.1 ± 8.8	117.92 ± 6.9	118.1 ± 9.9	0.941
Diastolic BP (mmHg)	73.0 ± 8.1	74.8 ± 5.5	72.1 ± 9.1	0.289
**Biochemical markers**				
Glucose	99.4 ± 13.1	103.3 ± 18.0	97.4 ± 9.6	0.308
Total cholesterol (mg/dL)	208.0 ± 36.4	200.8 ± 44.4	211.7 ± 32.0	0.457
Triglyceride (mg/dL)	101.8 ± 43.7	86.8 ± 26.1	109.7 ± 49.2	0.217
HDL (mg/dL)	71.2 ± 14.0	73.9 ± 12.6	69.8 ± 14.8	0.398
LDL (mg/dL)	116.3 ± 32.6	109.3 ± 39.8	120.0 ± 28.5	0.419
**Bone metabolic markers**				
Calcium (mg/dL)	9.7 ± 0.3	9.6 ± 0.2	9.8 ± 0.3	0.080
PTH (pg/mL)	30.3 ± 11.3	32.3 ± 11.3	29.3 ± 11.4	0.461
25-OH-Vitamin D (ng/mL)	34.0 ± 13.5	30.2 ± 13.6	35.9 ± 13.3	0.248
Serum osteocalcin (ng/mL)	13.8 ± 5.3	11.9 ± 3.3	14.8 ± 6.0	0.130
Urine NTx (mMBCE/mM Cr)	55.8 ± 19.5	53.3 ± 17.3	57.1 ± 20.7	0.575
**Bone Mineral Density (BMD)**				
Femur neck BMD (g/cm^2^)	0.856 ± 0.106	0.838 ± 0.100	0.866 ± 0.110	0.447
Femur total BMD (g/cm^2^)	0.890 ± 0.099	0.868 ± 0.075	0.901 ± 0.109	0.294
Lumbar BMD (g/cm^2^)	1.057 ± 0.127	1.025 ± 0.113	1.074 ± 0.132	0.327
**Lifestyle**				
Regular aerobic exercise, Yes (*n*)	24	9	15	0.835
Exercise time/week (min)	157.7 ± 147.3	158.3 ± 119.2	157.4 ± 162.5	0.986
Alcohol drinking, Yes (*n*)	7	4	3	0.327
Alcohol amount, 50cc/week	2.1 ± 8.6	5.2 ± 14.2	0.5 ± 1.9	0.119

BMI, body mass index; BP, blood pressure; HDL, high-density lipoprotein; LDL, low-density lipoprotein; PTH, parathyroid hormone; NTx, *N*-telopeptide.

**Table 2:** Storage stability of calcium and vitamin D in the study diet HMR.

	Calcium (mg/Pack)		Vitamin D (μg/Pack)	
Period (Month)	Control	Treatment	Control	Treatment
Baseline	9.720 ± 0.818	417.6 ± 15.28	ND	20.92 ± 1.024
6	9.300 ± 0.472	410.8 ± 14.70		20.73 ± 0.727
12	6.720 ± 0.370	400.9 ± 13.97		19.09 ± 0.557
18	6.000 ± 0.663	398.8 ± 13.72		18.15 ± 0.855

Values are mean ± SD, ND: not detected. Calcium and vitamin D in the study diet were determined in triplicate by ICP-MS and LC-MS/MS.

**Table 3:** Influence of usual exercise status on 6-month changes in bone mineral density and metabolic markers in the treatment group.

Variables	Regular Exercise (*n* = 15)	Lack of Exercise (*n* = 8)	*p*-Value
**Bone mineral density (BMD)**			
Δ Femur neck BMD (g/cm^2^)	−0.010 ± 0.033	0.003 ± 0.030	0.375
Δ Femur total BMD (g/cm^2^)	0.064 ± 0.257	0.007 ± 0.025	0.723
Δ Lumbar BMD (g/cm^2^)	0.004 ± 0.017	−0.013 ± 0.015	0.028
**Bone metabolic biomarker**			
Δ PTH (pg/mL)	3.5 ± 15.3	−0.3 ± 12.4	0.529
Δ 25-OH-Vitamin D (ng/mL)	1.1 ± 10.5	−3.0 ± 14.0	0.483
Δ Osteocalcin(ng/mL)	−1.2 ± 7.9	−0.3 ± 5.2	1.000
Δ NTx	2.5 ± 18.5	2.5 ± 20.2	0.897
Δ Urine calcium/creatinine	0.0 ± 0.0	0.0 ± 0.1	0.205

PTH, parathyroid hormone; NTx, *N*-telopeptide. Δ indicates the change value (difference between baseline and post-intervention). Regular exercise was defined as performing weight-bearing aerobic exercise for at least 90 min per week prior to the study, while lack of exercise was defined as 0 min per week.

**Table 4:** Comparison of changes in bone mineral density and bone metabolic markers by habitual exercise status among osteopenic participants: control vs. treatment group.

Variables	Regular Exercise	Lack of Exercise	*p*-Value
**Control**	**(*n* = 6)**	**(*n* = 2)**	
**Bone mineral density (BMD)**			
Δ Femur neck BMD (g/cm^2^)	0.012 ± 0.022	0.010 ± 0.006	0.857
Δ Femur total BMD (g/cm^2^)	−0.002 ± 0.026	−0.021 ± 0.025	0.314
Δ Lumbar BMD (g/cm^2^)	0.007 ± 0.023	0.000 ± 0.033	0.867
**Bone metabolic biomarker**			
Δ PTH (pg/mL)	5.167 ± 17.555	5.500 ± 0.707	1.000
Δ 25-OH-Vitamin D (ng/mL)	4.500 ± 12.808	−1.450 ± 1.202	0.429
Δ Osteocalcin(ng/mL)	−1.300 ± 7.365	3.150 ± 5.586	0.429
Δ NTx	−3.167 ± 15.198	−21.000 ± 24.042	0.241
Δ Urine calcium/creatinine	−0.033 ± 0.134	0.000 ± 0.085	1.000
**Treatment**	**(*n* = 7)**	**(*n* = 6)**	
**Bone mineral density (BMD)**			
Δ Femur neck BMD (g/cm^2^)	−0.009 ± 0.038	0.008 ± 0.029	0.386
Δ Femur total BMD (g/cm^2^)	0.129 ± 0.381	0.006 ± 0.023	0.721
Δ Lumbar BMD (g/cm^2^)	0.009 ± 0.006	−0.018 ± 0.012	0.001
**Bone metabolic biomarker**			
Δ PTH (pg/mL)	10.143 ± 16.965	−0.500 ± 14.612	0.250
Δ 25-OH-Vitamin D (ng/mL)	−0.700 ± 10.997	−5.533 ± 14.106	0.513
Δ Osteocalcin(ng/mL)	−1.543 ± 11.074	0.133 ± 5.247	0.886
Δ NTx	9.143 ± 22.901	2.167 ± 22.903	0.595
Δ Urine calcium/creatinine	0.023 ± 0.052	0.013 ± 0.075	0.800

PTH, parathyroid hormone; NTx, *N*-telopeptide. Δ indicates the change value (difference between baseline and post-intervention). Regular exercise was defined as performing weight-bearing aerobic exercise for at least 90 min per week prior to the study, while lack of exercise was defined as 0 min per week.

## Data Availability

The raw data supporting the conclusions of this article will be made available by the authors on request.

## Supplementary Materials

The following supporting information can be downloaded at: https://www.mdpi.com/article/10.3390/nu18040605/s1, Table S1: Changes in bone mineral density and bone metabolic markers after a 6-month intervention.; Table S2: Influence of usual exercise status on 6-month changes in bone mineral density and metabolic markers in the control group.

## Abbreviations

The following abbreviations are used in this manuscript:

ALT	Alanine aminotransferase
AST	Aspartate aminotransferase
AJOUIRB	Ajou University Institutional Review Board
BMD	Bone mineral density
BMI	Body mass index
Ca/Cr	Urinary calcium-to-creatinine ratio
DXA	Dual-energy X-ray absorptiometry
FSH	Follicle-stimulating hormone
HbA1c	Hemoglobin A1c
HDL	High-density lipoprotein
HMR	Home meal replacement
IRB	Institutional Review Board
LDL	Low-density lipoprotein
NTx	*N*-terminal telopeptide of type I collagen
PTH	Parathyroid hormone
PP	Per-protocol
RCT	Randomized controlled trial
SD	Standard deviation
WHO	World Health Organization
25(OH)D	25-hydroxyvitamin D
