# Supplementary material for: Effects of Eggshell Calcium- and Vitamin D-Fortified HMR Combined with Aerobic Exercise on Bone Mineral Density in Postmenopausal Women: A Pilot Randomized Controlled Trial
Source: Nutrients. 2026 Feb 12;18(4):605. doi: 10.3390/nu18040605 (PMC12942995; doi:10.3390/nu18040605)
Supplement: Supplementary file 1 [file nutrients-18-00605-s001.zip › nutrients-4120481-supplementary.pdf]

Table S1. Changes of bone mineral density and bone metabolic markers after a 6-month intervention.

Variables	Control (n=12)	Treatment (n=23)	*p*-Value
**Bone mineral density (BMD)**			
Δ Femur neck BMD (g/cm²)	-0.004 ± 0.032	-0.006 ± 0.032	0.901
Δ Femur total BMD (g/cm²)	-0.006 ± 0.023	0.045 ± 0.208	0.154
Δ Lumbar BMD (g/cm²)	0.001 ± 0.026	-0.002 ± 0.018	0.704
**Bone metabolic biomarker**			
Δ PTH (pg/ml)	5.3 ± 13.4	2.217 ± 14.161	0.539
Δ25-OH-Vitamin D (ng/mL)	-0.1 ± 10.6	-0.3 ± 11.7	0.959
Δ Osteocalcin(ng/mL)	-0.6 ± 5.6	-0.883 ± 7.0	0.889
Δ NTx	-5.7 ± 16.2	2.5 ± 18.6	0.375
Δ Urine calcium/creatinine	-0.1 ± 0.1	0.0 ± 0.1	0.085

PTH, parathyroid hormone; NTx, *N*-telopeptide.

Table S2. Influence of usual exercise status on 6-month changes in bone mineral density and metabolic markers in the control group.

Variables	Regular exercise (n=9)	Lack of exercise (n=3)	*p*-Value
**Bone mineral density (BMD)**			
Δ Femur neck BMD (g/cm²)	-0.005 ± 0.036	-0.001 ± 0.019	0.843
Δ Femur total BMD (g/cm²)	-0.006 ± 0.022	-0.007 ± 0.030	0.962
Δ Lumbar BMD (g/cm²)	0.001 ± 0.028	0.000 ± 0.024	0.962
**Bone metabolic biomarker**			
Δ PTH (pg/ml)	4.0 ± 15.2	9.0 ± 6.1	0.601
Δ 25-OH-Vitamin D (ng/mL)	0.3 ± 12.4	-1.2 ± 0.9	0.839
Δ Osteocalcin(ng/mL)	-1.4 ± 5.9	1.7 ± 4.7	0.436
Δ NTx	-0.4 ± 12.9	-21.3 ± 17.0	0.063
Δ Urine calcium/creatinine	-0.1 ± 0.1	-0.0 ± 0.1	0.853

PTH, parathyroid hormone; NTx, *N*-telopeptide.
